# The Unknown Benefits of Dermal Matrices in Prevent Loss of Albumin in Patient with Major Burns

**DOI:** 10.3390/ebj7030048

**Published:** 2026-09-17

**Authors:** Wen Ching Jeannette Ting, Long Yin Ronald Cheung, Hui Ma, Aydin Kerem Arslan, Jun Fung Max Kam, Tor Wo Chiu

**Affiliations:** Division of Plastic, Reconstructive and Aesthetic Surgery, Department of Surgery, Prince of Wales Hospital, The Chinese University of Hong Kong, Hong Kong SAR, China; ronaldcheung@surgery.cuhk.edu.hk (L.Y.R.C.); huima@cuhk.edu.hk (H.M.); a.arslan@ogr.iu.edu.tr (A.K.A.); kam.maximillian@gmail.com (J.F.M.K.); torchiu@surgery.cuhk.edu.hk (T.W.C.)

**Keywords:** major burns, albumin, hypoalbuminaemia, dermal matrix

## Abstract

**Highlights:**

**What are the main findings?**

- BTM was associated with significantly faster albumin recovery compared to conventional skin grafting, with notable intergroup differences observed at weeks 5–7.
- Sustained recovery was observed exclusively in the BTM group from week 3 onward.

**What are the implications of the main findings?**

- BTM may help reduce hypoalbuminemia-related complications (e.g., infection, edema, delayed healing) in major burn patients.
- Beyond wound coverage, BTM may serve as a metabolic support tool, guiding nutritional and recovery protocols in burn care.

**Abstract:**

**Background:** Hypoalbuminemia is a common complication after burn injury, exacerbated by surgical debridement, and contributes to edema, delayed healing, infection, and mortality. NovoSorb^®^ biodegradable temporizing matrix (BTM) is a dermal substitute that may reduce protein loss from open wounds. **Objective:** To evaluate the effect of BTM on serum albumin levels compared with conventional split-thickness skin grafting. **Method:** This retrospective review included burn patients (TBSA ≥ 10%, length of stay > 30 days) admitted between 2020 and 2025 who underwent debridement followed by BTM or skin grafting. Serial albumin levels were analyzed as weekly means. **Result:** Baseline characteristics were comparable between BTM (*n* = 14) and non-BTM (*n* = 16) groups. Albumin decreased in both groups at week 1. From week 3 onward, albumin levels rose only in the BTM group, with significant intergroup differences at weeks 5–7 (*p* < 0.05 to *p* < 0.01), but not at week 8. **Conclusions:** In this observational, exploratory, and hypothesis-generating study, BTM application was associated with faster albumin recovery compared with conventional methods. This is the first study to suggest that a dermal substitute may positively influence systemic albumin dynamics in burn patients, highlighting its potential role in reducing protein loss and supporting metabolic recovery. However, given the preliminary nature of these findings, it requires confirmation in larger, prospective, randomized controlled trials.

## 1. Introduction

Hypoalbuminemia is one of the earliest and most important complications following a major burn. This is primarily attributable to the triggering of a severe inflammatory cascade, which leads to enhanced capillary permeability and extensive plasma protein leakage into the interstitial space [1,2]. It is further aggravated by surgical debridement and grafting, where patients are left with even larger raw surfaces, leading to further albumin loss. Albumin is vital in preserving oncotic pressure, delivering antioxidants, and regulating inflammation [1]. As a result, patients with hypoalbuminemia suffer from interstitial oedema, delayed wound healing, and multi-organ dysfunction. Research suggests that carefully timed albumin therapy, especially after the first 24 h, may support hemodynamic stability and reduce total fluid requirements [3]. However, albumin replacement therapy has historically been controversial due to its high cost, potential for allergic reactions, and concerns about intravascular leakage and, therefore, its efficacy.

NovoSorb^®^ biodegradable temporizing matrix (BTM) is a synthetic polyurethane dermal substitute that has been used to temporarily cover large burn wounds [4]. It is a bilaminar structure composed of an open-cell foam scaffold that allows for ingrowth of neodermis that is covered by a non-biodegradable temporary sealing membrane. This study hypothesizes that BTM may be able to seal burn wounds, thus preventing albumin loss whilst the patient undergoes staged operations and physiologically recovers from their major burns.

To date, clinical studies on dermal substitutes, including NovoSorb^®^ BTM, have primarily focused on local outcomes such as wound closure rates, graft take percentage, incidence of infection, and scar quality. However, the systemic physiological effects of these matrices, particularly their impact on the hypermetabolic response and nutritional parameters like serum albumin, remain largely unexplored. Unlike biological or xenogeneic substitutes, synthetic biodegradable matrices may modulate the inflammatory burden differently, potentially conferring systemic benefits that extend beyond simple wound coverage. Therefore, in this study, we aimed to evaluate whether NovoSorb^®^ BTM offers an additional, previously unrecognized advantage, supporting serum albumin recovery, compared with conventional split-thickness skin grafting in patients with major burns.

## 2. Materials and Methods

A retrospective cohort study was conducted on patients admitted to the Burn Units of Prince of Wales Hospital between January 2020 and December 2025. Eligible patients were those who sustained burns covering ≥10% of total body surface area (TBSA), had a hospital length of stay exceeding 30 days, and underwent surgical debridement of burn wounds followed by either application of BTM or split-thickness skin grafting (non-BTM group). The standard protocol for NovoSorb^®^ BTM (PolyNovo Biomaterials Pty Ltd., Port Melbourne, Victoria, Australia) follows a two-stage process. After meticulous wound debridement, washout, and hemostasis, BTM is cut to size, placed foam-side down onto the wound bed, and secured with staples or sutures. The sealing membrane remains intact during a 4–6-week integration period, during which wound dressings are regularly performed. Once vascularization is confirmed, the sealing membrane is delaminated, leaving a vascularized neodermis ready for split-thickness skin grafting. The SSG protocol involves harvesting a partial-thickness skin graft from a donor site (typically the thigh) using a dermatome. The graft is then meshed if required, placed onto the prepared wound bed, and secured with sutures or staples. A bolster or negative pressure dressing is often applied to secure the graft in place, and the donor site is dressed to heal by re-epithelialization.

The following data were extracted from medical records. Baseline parameters included demographic characteristics, mechanism of injury, percentage TBSA burned, and time from injury to surgical intervention. Primary outcomes included serial serum albumin levels measured at predefined time points. Serum albumin levels were analyzed as mean values per post-injury week for each patient group. Secondary outcomes comprised exogenous albumin administration, presence of infection, number of operating room (OR) trips, graft take, and discharge status.

The data were expressed as mean ± standard deviation (SD). All data were subjected to *t*-test or two-way ANOVA using GraphPad Prism 5 (San Diego, CA, USA). Bonferroni tests were applied as post hoc comparisons for two-way ANOVA. A *p* value of less than 0.05 was considered statistically significant.

Post hoc power analysis was conducted using G*Power (v3.1.9.7, Heinrich Heine University Düsseldorf, Düsseldorf, Germany) for the primary endpoint of serum albumin levels at weeks 5, 6, and 7. Observed power was calculated using an independent two-sample *t*-test (α = 0.05, two-tailed), with effect sizes derived from observed means, pooled standard deviations, and sample sizes at each time point. Sensitivity analyses determined the minimum detectable effect size for 80% power, and sample size estimates for future studies were calculated using the observed effect sizes with adjustments for attrition.

## 3. Results

### 3.1. Patient Baseline Characteristics

**Figure 1:**
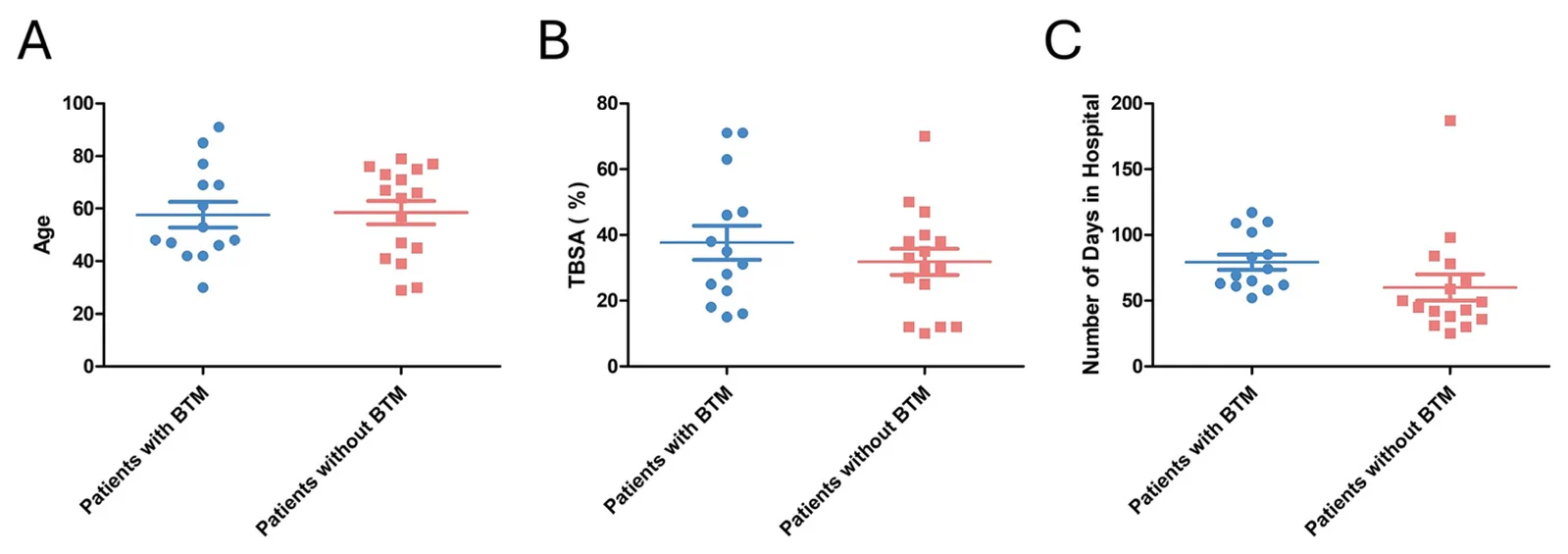
Comparison of baseline characteristics between the BTM (*n* = 14) and non-BTM (*n* = 16) groups. (**A**) Age (years), (**B**) TBSA (%), and (**C**) length of hospital stay (days). No significant differences were found between groups (*p* > 0.05 for all). Data are shown as mean ± SD or percentages.

A total of 30 patients were included in the study, with 14 in the BTM group and 16 in the non-BTM group. They were homogeneous. In the BTM group, there were five females and nine males, compared to nine females and seven males in the non-BTM group. Baseline characteristics were comparable between the BTM and non-BTM groups. The mean age was 57.7 years (95% CI: 48.3–67.1) in the BTM group and 58.5 years (95% CI: 50.0–67.0) in the non-BTM group (*p* = 0.90). Mean TBSA burned was 38% (95% CI: 28–47%) in the BTM group and 32% (95% CI: 24–40%) in the non-BTM group (*p* = 0.38). Mean length of hospital stay was 79.3 days (95% CI: 67.8–90.8) in the BTM group and 60.0 days (95% CI: 40.5–79.5) in the non-BTM group (*p* = 0.12). Burn mechanism was also similar between groups: flame burns occurred in 71% of BTM patients and 75% of non-BTM patients, while scald burns accounted for 29% and 25%, respectively. Among BTM patients, the mean time from admission to BTM application was 10.0 days (95% CI: 6.4–13.6) (Figure 1A–C) (Table 1 and Table 2).

### 3.2. Primary Outcomes: Mean Serum Albumin Levels

**Figure 2:**
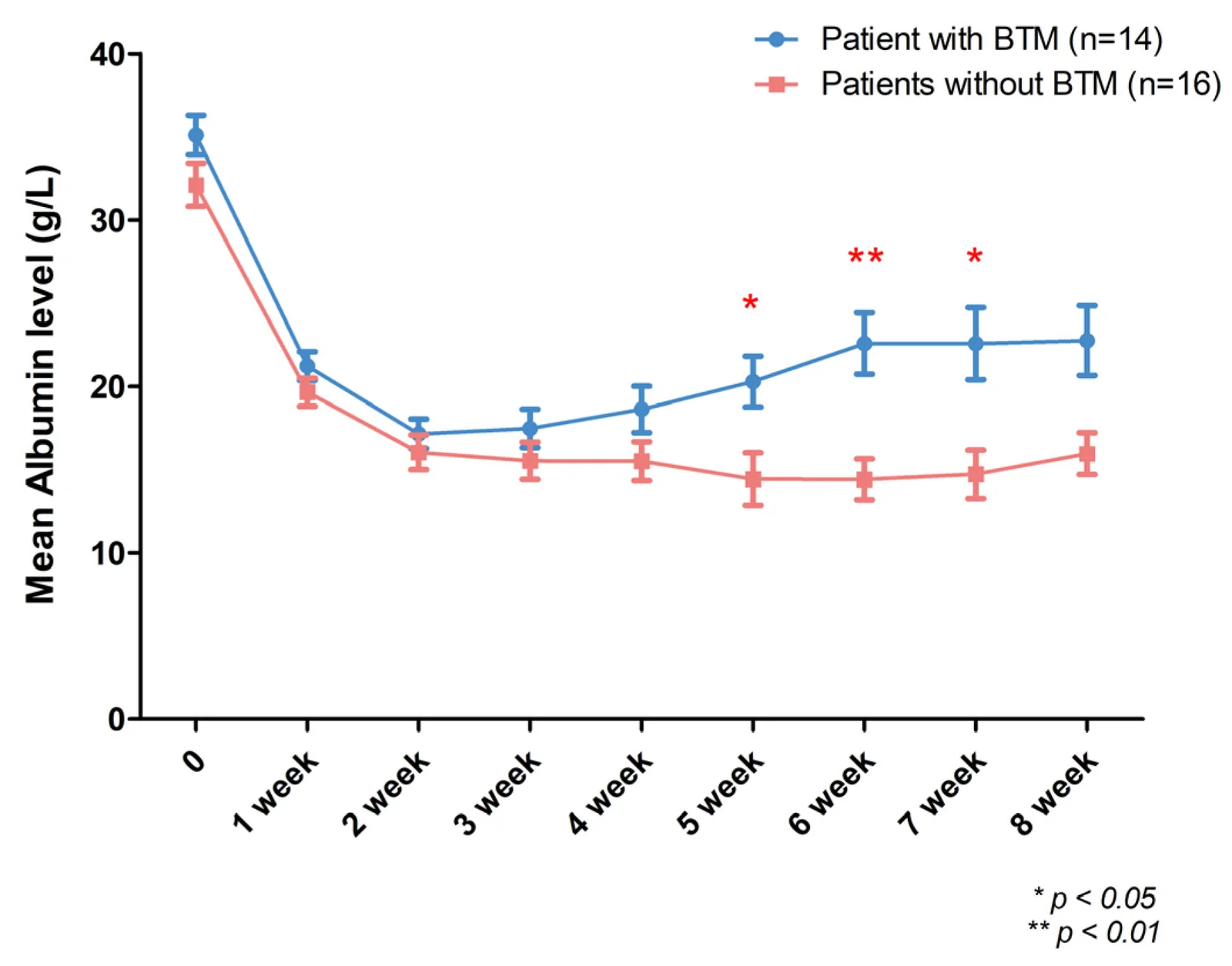
Comparison of weekly mean serum albumin levels between BTM (*n* = 14) and non-BTM (*n* = 16) groups from admission to week 8.

Serum albumin levels were compared weekly. At baseline (admission), mean albumin levels did not differ significantly between the two groups. One week post-admission, both groups exhibited a marked decrease in albumin levels. From week 3 onward, mean albumin levels began to increase only in the BTM group. Statistically significant differences between the groups were observed from week 5 to week 7 (week 5: *p* < 0.05; week 6: *p* < 0.01; week 7: *p* < 0.05). No statistical difference was observed at week 8 (Figure 2).

At week 5, the study had 73.5% power to detect the observed difference (Cohen’s d = 1.19, *p* = 0.016). At week 6, observed power was 78.0% (Cohen’s d = 1.46, *p* = 0.010). At week 7, observed power declined to 58.8% (Cohen’s d = 1.36, *p* = 0.044), largely attributable to patient attrition reducing the size of the non-BTM group at this time point. Sensitivity analysis indicated that, with the available sample sizes, the study could detect a minimum effect size of d = 1.19 at week 5, d = 1.32 at week 6, and d = 1.72 at week 7 with 80% power. This confirms that the study was adequately powered to detect large effect sizes but was underpowered for smaller, potentially meaningful differences, particularly at later time points. Based on the observed effect sizes from this study, a priori sample size calculations for future prospective trials aiming for 80% power would require approximately 16–20 patients per group to account for around 30% patient attrition at later time points.

### 3.3. Secondary Outcomes

All patients received standardized nutritional protocols in accordance with institutional guidelines. The mean daily exogenous albumin administered was similar between groups (BTM: 2.13 g/day vs. non-BTM: 1.96 g/day; *p* = 0.85). Infection was present in 13 of 14 patients (92.9%) in the BTM group and 15 of 16 patients (93.8%) in the non-BTM group. All patients underwent OR debridement. The number of OR debridement procedures and total OR trips were also comparable between groups. Mean OR debridement episodes were 1.29 in the BTM group vs. 1.81 in the non-BTM group (*p* = 0.15), while total OR trips were 3.64 vs. 2.75, respectively (*p* = 0.08). The BTM group required a higher number of OR trips due to the necessity of a two-stage application process. Complete graft take was achieved in all patients in both groups. All patients were discharged home, except for two deaths after the study period in the BTM group, both attributable to extensive TBSA burns and comorbidities.

## 4. Discussion

Albumin levels are a reliable indicator of prognosis. Serum albumin values below 30 g/L on admission are associated with and result in increased risk of sepsis, delayed wound healing, longer ventilatory support, and increased mortality [5]. Within hours of a severe burn, the vascular endothelium’s integrity is weakened, leading to significant albumin loss from burn wound exudation and vascular leakage [6]. The persistence of capillary dysfunction in the early post-burn phase was highlighted by Zdolsek et al., who clinically showed that albumin escape rates remained abnormally elevated for several hours even after 20% human albumin was administered to burnt patients [6]. This leakage persists for up to 24 to 36 h following injury, or until capillary integrity is restored [7]. Exudative loss is correlated with wound surface exposure, TBSA, and burn depth. These losses may be exacerbated by open wound management techniques and postponements of early excision and grafting [8]. Although intravenous albumin is commonly administered to maintain serum albumin levels, its clinical efficacy beyond maintaining blood pressure levels remains controversial.

Surgically, definitive coverage of wounds by skin grafts has been shown to improve albumin levels, albeit slowly [9]. Grafts may also be classified according to their origin. Autografts, harvested from the patient’s own skin, are the standard approach for permanent wound coverage in burn patients. However, patients with large total body surface area burns may have very limited donor sites that require complete healing before they can be reused. In contrast, cadaveric allografts, derived from human donors, serve as temporary biological dressings to temporize the wound bed for subsequent autografting. They are subject to immune rejection but provide a valuable bridging strategy during the interval required for donor site healing [10].

Dermal matrices have been used extensively in burns to temporize wounds when donor sites for skin grafts are limited. Studies in the literature show that dermal matrices can be used effectively to manage full-thickness burns with good functional and aesthetic outcomes [11,12]. However, dermal matrices are associated with a notable risk of infection, with one study having to stop due to the high infection rate associated with the use of Integra^®^ (Integra Artificial Skin, Integra Life Sciences, Plainsboro, NJ, USA) in patients with major burns [13]. Integra is made from a combination of bovine collagen with cross-linked glycosaminoglycans, and it is thought that the biological component is what increases its risk of infection. BTM is different from other dermal matrices in that it lacks a biological component and is completely synthetic in composition. Systematic reviews have shown that the infection rate in over 800 patients who used BTM averaged 10% in complex wounds [14]. BTM has also been used successfully in infected and contaminated wounds [15,16]. Its uptake rate in burns can be as high as over 88% compared to the skin graft take rate of 82% [12]. Second-stage graft take has been shown in a systematic review to be over 98.9% in nine studies of 511 wounds. Integra^®^, by comparison, has reported graft take rates between 90% and 93% [17]. While previous investigations of dermal matrices in burns have reported encouraging local outcomes, including durable wound closure and acceptable graft take, our findings extend this knowledge by revealing a potential systemic metabolic benefit. Specifically, we observed in our case-matched patients that there is a significant difference in albumin levels in patients with major burns between weeks 5 and 7 post-injury. BTM application averaged 10 days post-injury, which can be reflected in the beginning of the divergence of albumin levels between patients who had BTM coverage and those who did not. Also, the albumin levels in patients who did not have BTM stabilized, whereas the albumin levels in patients with BTM continued to elevate. This supports our hypothesis that reconstructive dermal matrices can improve albumin levels and therefore patient recovery, suggesting that the BTM may do more than provide a physical scaffold; it may actively mitigate the persistent protein loss and inflammatory catabolism that characterize the hypermetabolic phase of major burns.

To our knowledge, this is the first study to report an association between a synthetic biodegradable temporizing matrix and systemic albumin recovery in major burn patients. This finding is clinically relevant for several reasons. First, hypoalbuminemia is a robust predictor of morbidity and mortality in burns, yet current management strategies rely largely on exogenous supplementation and nutritional support, which are often insufficient. Second, our data raise the hypothesis that early application of BTM may reduce the duration and intensity of the systemic inflammatory response by providing rapid, stable wound coverage with reduced antigenic and infectious stimuli, thereby preserving endogenous albumin synthesis. Third, this work shifts the paradigm for evaluating dermal substitutes from purely local wound-healing metrics to a broader assessment of systemic recovery. We acknowledge that this is an observational finding and requires mechanistic validation; nevertheless, it opens a new avenue for research into the systemic effects of synthetic scaffolds in critical illness.

The observed improvement in serum albumin recovery in the BTM group carries potentially significant clinical implications. Restoration of albumin may directly reduce interstitial oedema, thereby improving tissue oxygenation, facilitating nutrient delivery to healing wounds, and decreasing the risk of compartment syndromes [18]. Furthermore, higher circulating albumin levels are associated with improved leukocyte functions, enhanced bacterial clearance, and reduced susceptibility to wound infections [19]. Given that infection remains a leading cause of morbidity in major burns, even modest improvements in albumin status could translate into meaningful reductions in septic complications. Faster albumin recovery may also influence important clinical endpoints such as ICU length of stay, duration of mechanical ventilation, and mortality. Hypoalbuminemia is an independent predictor of prolonged ventilatory support and increased ICU resource utilization [19]. By mitigating the inflammatory burden and reducing protein loss, BTM may contribute to earlier hemodynamic stabilization and weaning from organ support.

Of note, there was no significant difference in the length of hospital stay between patients who had BTM and those who did not. There are myriad variables associated with length of stay in hospital after burn injury. These include socio-economic reasons, discharge destination, and concurrent medical issues that cannot be compared.

Nevertheless, several limitations of this study should be acknowledged. Foremost among these is the relatively small sample size (*n* = 30 total, with 14 in the BTM group and 16 in the non-BTM group), which limits the statistical power of our analysis. As this was an exploratory study in a challenging burn population, a formal a priori sample size calculation was not performed. Post hoc power analysis for albumin recovery revealed observed powers of 73.5% (week 5, d = 1.19), 78.0% (week 6, d = 1.46), and 58.8% (week 7, d = 1.36), with the decline at week 7 attributable to patient attrition. Sensitivity analysis confirmed adequate power for large effect sizes but insufficient power for smaller differences. The modest sample size limits generalizability and underscores the need for larger prospective studies.

Second, the retrospective design and absence of case-matched pairing introduce potential selection bias. While all patients received standardized nutritional and resuscitation protocols per institutional guidelines, we cannot exclude the possibility that unmeasured variables influenced our results. This limitation underscores the need for prospective studies with a systematic collection of relevant confounders to confirm our findings.

Third, our study was not designed or powered to detect differences in clinically meaningful endpoints such as ICU length of stay, duration of mechanical ventilation, or mortality; therefore, any discussion of these potential benefits remains speculative and hypothesis-generating.

Fourth, our study lacked long-term follow-up data. The serum albumin levels of patients remained below 30 g/L at the predefined cutoff time point, suggesting that persistent hypoalbuminemia may have continued to influence patient physiology beyond our observation window. Consequently, a longer period of follow-up would be valuable to capture more definitive clinical endpoints, such as overall mortality and total length of hospital stay, and to assess patient-centered outcomes, including scar quality, functional outcomes, quality of life, and late complications such as contractures or reconstructive procedures. The durability of the observed albumin recovery and its relationship to these long-term outcomes therefore remain unknown.

These limitations should be taken into consideration when interpreting our results. In particular, the small sample size and lack of matching underscore the need for future prospective, randomized controlled trials with adequate statistical power and predefined matching criteria to confirm whether the observed albumin-sparing effect of BTM is reproducible and clinically meaningful.

## 5. Conclusions

In conclusion, this observational, exploratory, and hypothesis-generating study suggests that NovoSorb^®^ BTM not only provides effective temporary wound coverage but may also confer a novel systemic benefit by facilitating serum albumin recovery in major burn patients. This finding challenges the conventional view of dermal substitutes as purely local therapies and underscores the need for future studies to explore their immunometabolic effects. If confirmed in larger prospective trials, the albumin-sparing effect of BTM could translate into clinically meaningful benefits, including reduced infectious complications, shorter ICU and hospital stay, and potentially improved survival. However, we caution against overinterpreting our findings. As our study was not powered to detect differences in these hard clinical endpoints, the small sample size and lack of case matching limit the strength of our conclusions. Furthermore, the long-term implications of improved early albumin recovery remain unexplored and represent a critical gap in the current evidence base. Prospective, randomized controlled trials with larger sample sizes, predefined matching criteria, standardized protocols, and extended follow-up periods are urgently needed to validate these observations and to determine whether the systemic benefits of BTM ultimately improve patient-centered outcomes in major burn care.

## Figures and Tables

**Table 1:** Information of patients.

Patient No.	Treatment	Gender	Age	TBSA (%)	Days in Hospital	BTM Day (Post Admission)	Mechanisms
1	BTM	F	85	28%	83	D11	Scald
2	BTM	F	46	47%	110	D5	Flame
3	BTM	F	42	25%	62	D5	Flame
4	BTM	F	91	15%	117	D10	Scald
5	BTM	F	47	18%	58	D31	Flame
6	BTM	M	77	35%	85	D11	Scald
7	BTM	M	69	63%	74	D6	Flame
8	BTM	M	61	38%	69	D6/12	Flame
9	BTM	M	48	46%	109	D3/26	Flame
10	BTM	M	48	16%	61	D11	Scald
11	BTM	M	53	71%	65	D4/8	Flame
12	BTM	M	69	31%	102	D6	Flame
13	BTM	M	42	23%	63	D13	Flame
14	BTM	M	30	71%	52	D2	Flame
15	Non-BTM	F	67	12%	25	N.A.	Flame
16	Non-BTM	F	47	12%	42	N.A.	Flame
17	Non-BTM	F	73	10%	36	N.A.	Flame
18	Non-BTM	F	77	38%	65	N.A.	Scald
19	Non-BTM	F	75	30%	187	N.A.	Scald
20	Non-BTM	F	66	27%	30	N.A.	Scald
21	Non-BTM	F	76	12%	31	N.A.	Scald
22	Non-BTM	F	45	33%	84	N.A.	Flame
23	Non-BTM	F	41	47%	50	N.A.	Flame
24	Non-BTM	M	39	25%	49	N.A.	Flame
25	Non-BTM	M	79	38%	45	N.A.	Flame
26	Non-BTM	M	71	35%	98	N.A.	Flame
27	Non-BTM	M	29	70%	38	N.A.	Flame
28	Non-BTM	M	64	30%	43	N.A.	Flame
29	Non-BTM	M	57	40%	78	N.A.	Flame
30	Non-BTM	M	30	50%	59	N.A.	Flame

**Table 2:** Comparison of baseline characteristics in BTM and non-BTM patients.

Characteristics	BTM (*n* = 14)	Non-BTM (*n* = 16)	*p* Value
Age (years), mean (95% CI)	57.7 ± 18.0 (48.3–67.1)	58.5 ± 17.4 (50.0–67.0)	0.9
TBSA (%), mean (95% CI)	38 ± 19 (28–47)	32 ± 16 (24–40)	0.38
Length of hospital stay (days), mean (95% CI)	79.3 ± 21.9 (67.8–90.8)	60.0 ± 39.7 (40.5–79.5)	0.12
Burn mechanism, *n* (%)			
Flame	10 (71)	12 (75)	—
Scald	4 (29)	4 (25)	—
Time from admission to BTM application (days), mean (95%CI)	10.0 ± 7.8 (6.4–13.6)	—	—
Time from admission to SSG application (days), mean (95% CI)	46.2 ± 20.8 (57.2–35.2)	8.3 ± 6.8 (11.7–4.8)	<0.001

## Data Availability

The data presented in this study are available upon request from the corresponding author due to ethical reasons.

## Abbreviations

The following abbreviations are used in this manuscript:

BTM	Biodegradable temporizing matrix
OR	Operation room
TBSA	Total body surface area
SD	Standard deviation
TNF-α	Tumor necrosis factor-alpha
IL-1	Interleukin-1
IL-6	Interleukin-6
